# Sub-oxycline methane oxidation can fully uptake CH_4_ produced in sediments: case study of a lake in Siberia

**DOI:** 10.1038/s41598-020-60394-8

**Published:** 2020-02-25

**Authors:** Frédéric Thalasso, Armando Sepulveda-Jauregui, Laure Gandois, Karla Martinez-Cruz, Oscar Gerardo-Nieto, María S. Astorga-España, Roman Teisserenc, Céline Lavergne, Nikita Tananaev, Maialen Barret, Léa Cabrol

**Affiliations:** 1 Biotechnology and Bioengineering Department, Center for Research and Advanced Studies (Cinvestav), Mexico City, Mexico; 2 The Environmental Biogeochemistry in Extreme Ecosystems Laboratory (EnBEELab), University of Magallanes, Punta Arenas, Chile; 3 Center for Climate and Resilience Research (CR)2, Santiago, Chile; 4 EcoLab, Université de Toulouse, CNRS, Toulouse, France; 5 Escuela de Ingeniería Bioquímica, Pontificia Universidad de Valparaiso, Valparaiso, Chile; 6 Melnikov Permafrost Institute, Yakutsk, Russia; 7 Aix-Marseille University, Univ Toulon, CNRS, IRD, Mediterranean Institute of Oceanography, Marseille, France

**Keywords:** Freshwater ecology, Carbon cycle, Carbon cycle, Boreal ecology, Limnology

## Abstract

It is commonly assumed that methane (CH_4_) released by lakes into the atmosphere is mainly produced in anoxic sediment and transported by diffusion or ebullition through the water column to the surface of the lake. In contrast to that prevailing idea, it has been gradually established that the epilimnetic CH_4_ does not originate exclusively from sediments but is also locally produced or laterally transported from the littoral zone. Therefore, CH_4_ cycling in the epilimnion and the hypolimnion might not be as closely linked as previously thought. We utilized a high-resolution method used to determine dissolved CH_4_ concentration to analyze a Siberian lake in which epilimnetic and hypolimnetic CH_4_ cycles were fully segregated by a section of the water column where CH_4_ was not detected. This layer, with no detected CH_4_, was well below the oxycline and the photic zone and thus assumed to be anaerobic. However, on the basis of a diffusion-reaction model, molecular biology, and stable isotope analyses, we determined that this layer takes up all the CH_4_ produced in the sediments and the deepest section of the hypolimnion. We concluded that there was no CH_4_ exchange between the hypolimnion (dominated by methanotrophy and methanogenesis) and the epilimnion (dominated by methane lateral transport and/or oxic production), resulting in a vertically segregated lake internal CH_4_ cycle.

## Introduction

Methane (CH_4_) released from lakes to the atmosphere is generally assumed to be produced in anoxic sediment and transported by diffusion or ebullition through the water column^1,2^, where it is subject to oxidation, generally identified as a major sink^2–5^. In stratified lakes, the diffusion barrier formed by the thermocline promotes CH_4_ storage in the hypolimnion. The stored CH_4_ is released mainly to the epilimnion and then to the atmosphere during water column overturn^6,7^. Lateral transport of CH_4_ from the littoral to the pelagic zones may also substantially modify the CH_4_ balance at the epilimnion of the lakes^1,8,9^. Although this has been the prevailing theory, several other CH_4_ cycle processes have been discovered. For instance, CH_4_ production in the epilimnion under aerobic conditions has been observed in several aquatic ecosystems^8,10–13^. Another process previously reported is CH_4_ oxidation below the oxycline, which is typically associated with microaerophilic conditions^4^, oxygenic photosynthesis^4,14^, or, in some cases, assumed to be anaerobic^15^. These processes explain why deviations to standard CH_4_ concentration profiles (usually decreasing from the bottom to the surface) are often observed as concentrations increase or decrease in local areas.

In this study, we observed an atypical CH_4_ profile in a strongly stratified lake (Sila Lake) in north-central Siberia. From the bottom to the surface, the profile showed a sharp decrease of the CH_4_ concentration below the detection limit of our method (5 nmol L^−1^) in the benthic zone of the water column, well below the oxycline and the photic zone, and showed an increase in CH_4_ concentration in the aerobic epilimnion. This profile was created using a high resolution, high sensitivity method^16,17^ which allowed the net methane production and oxidation rates to be determined using a diffusion-reaction model, showing a short distance transition between both processes in the hypolimnion. This CH_4_ concentration profile was correlated to methanotroph and methanogenic archaea abundances determined by *pmoA* and *mcrA* gene qPCR, respectively.

## Results and Discussion

The depth measurements collected were used to develop a bathymetric map (Fig. S1) that shows a maximum depth of 12 m in the southeastern branch of the lake. Satellite imagery (Google Earth Engine) was used to determine that the area of the lake was 3.6 Ha. Combining the area of the lake and the bathymetric map, we constructed a hypsometric histogram to estimate the total water volume of the lake to be 168,000 m^3^. The lake was strongly stratified, with a mean surface temperature of 16.5 ± 0.7 °C (mean ± one standard deviation), a bottom temperature of 3.4 ± 0.3 °C, a thermocline 2–4 m deep, and a maximum gradient of 8.6 °C m^−1^ (Fig. S2). The oxycline matched the thermocline and anoxic conditions (i.e., dissolved oxygen (DO) concentration below 0.3 µmol L^−1^) were found at depths below 4 m. The Secchi depth was 1.8 ± 0.15 m, meaning that the euphotic depth (Z_1%_), i.e., depth at which the photosynthetically available radiation was 1% of its surface value, was estimated at 4.9 ± 0.4 to 5.6 ± 0.4 m.

**Figure 1:**
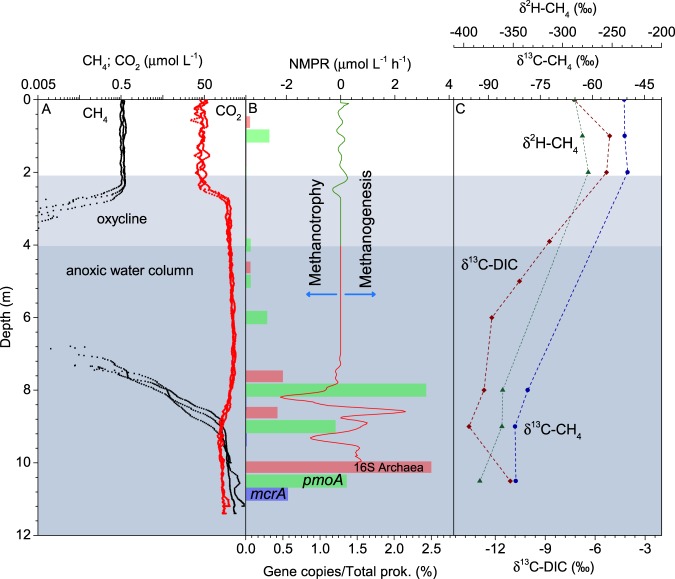
**(A)** Depth profiles of dissolved CH_4_ (C_CH4_) and CO_2_ (C_CO2_) in logarithmic scale; **(B)** net methane production rates of one profile (for clarity purposes, replicate profiles are shown in Fig. S3); and relative abundance, in reference to total prokaryotes, of pmoA genes (green bars), mcrA genes (blue bars) and total archaea genes (pink bars). Epilimnion NMPR are multiplied by 1000; **(C)** δ^13^C-DIC (brown square), δ^13^C-CH_4_ (green circles), and δ^2^H-CH_4_ (blue triangles).

**Figure 2:**
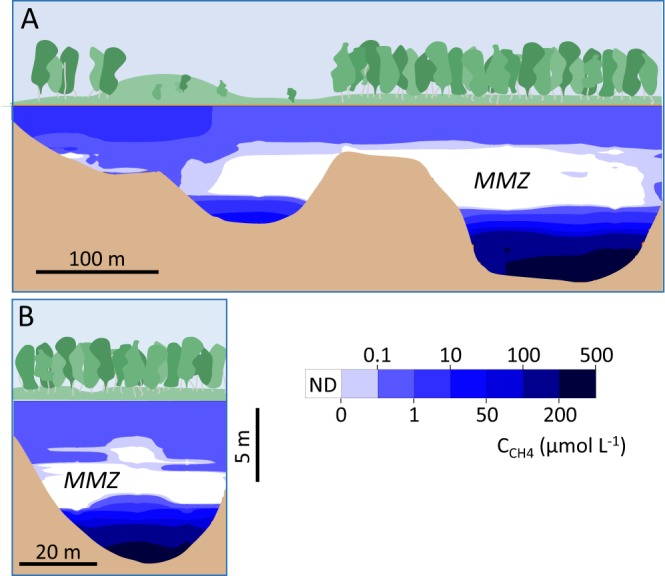
Longitudinal (east-west; **A**) and transversal (north-south; **B**) transectional maps of dissolved CH_4_ concentration showing the expansion of the minimum methane zone (MMZ). ND stands for not detected.

In addition to each sampling and monitoring station, the profiles of dissolved CH_4_ and carbon dioxide (CO_2_) concentration (C_CH4_ and C_CO2_, respectively) were measured in triplicate at the center of the lake (P1, see materials and methods, Fig. 1A). Epilimnetic C_CH4_ and C_CO2_, at depths between 0 and 2.5 m, were 0.56 ± 0.05 μmol L^−1^ and 44 ± 7 μmol L^−1^, respectively. Below 2.5 m, C_CH4_ rapidly decreased and was no longer detected below depths of 2.96 ± 0.13 m (until depths greater than 7 m). On the contrary, an increase of C_CO2_ was observed to 209 ± 16 μmol L^−1^. At a depth of 7.12 ± 0.14 m, well below the oxycline and Z_1%,_ C_CH4_ rapidly increased and reached 196 ± 18 μmol L^−1^ at 10 m. Higher values were observed below 10 m but these measurements were discarded as potentially being the result of sediment disturbances, at a depth of 10.4–10.7 m. Inversely, C_CO2_ decreased in the deepest section of the hypolimnion to 135 ± 11 μmol L^−1^ at 10 m. The section of the water column where CH_4_ was not detected (3–7 m), called the “methane minimum zone” (MMZ), expanded along the transversal and longitudinal transects (Fig. 2), and acted as a segregation zone between the hypolimnetic and the epilimnetic CH_4_.

To better characterize the CH_4_ profiles observed, a diffusion-reaction model was applied to estimate net methane production rates (NMPR; Fig. 1B, S3D–F) and vertical fluxes through the water column (Fig. S3A–C). As shown in the diagram, a peak of negative NMPR, i.e., CH_4_ sink, was observed below the MMZ, immediately above a peak of positive NMPR; i.e., CH_4_ source. The same pattern was observed in the three replicates (Fig. S3), although with differences in depths (maximum difference of 0.54 m between the three negative NMPR peaks), which were likely caused by boat motion and inaccurate positioning, evidenced by differences in water column depth varying between 10.4 and 10.7 m. It is noteworthy that the depth intervals between CH_4_ sink and source peaks were consistently small, 0.51 ± 0.11 m, indicating a sudden shift in the dominant CH_4_ process. The average minimum and maximum NMPR corresponding to the first methanotrophic and the methanogenic peaks, in a downward direction, were –1.82 ± 0.34 and 1.69 ± 0.68 μmol L^−1^ h^−1^, respectively, which suggests that the magnitude of both processes was comparable.

The isotopic signature of CH_4_ and the CH_4_ production fractionation factor (α; see materials and methods), show two homogeneous zones (Fig. 1C). In the hypolimnetic layer, depleted δ^13^C-CH_4_ (–78.6 to –82.2‰) and δ^2^H-CH_4_ (–360 to –383‰), coupled to relatively high α values (1.072 to 1.077), strongly support hydrogenotrophic production of CH_4_ from CO_2_^12,18^. On the contrary, at the epilimnion, enriched values of δ^13^C-CH_4_ (–49.8 to –50.8‰) and δ^2^H-CH_4_ (–274.2 to –287.8‰), coupled with relatively low α values (1.046 to 1.048), are consistent with a predominance of acetoclastic production of CH_4_^12,18^. Indeed, according to Whiticar and Faber^19^, α from 1.055 to 1.09 most likely corresponds to hydrogenotrophic methanogenesis, while α from 1.04 to 1.055 corresponds to acetoclastic methanogenesis.

The abundance of methane oxidizing bacteria (MOB) quantified through the relative abundance of *pmoA* gene (Fig. 1B) was minimal in the MMZ, representing <0.03% of the prokaryotic community, as expected from the absence of detectable levels of CH_4_. Higher abundance of MOB was detected within the anoxic water column (8–10 m), where MOB represented from 1.1% to 2.5% of the total community. Notably, the peak of MOB abundance was found at 8 m depth (1.6 ± 0.05 × 10^8^ *pmoA* copies L^−1^), which coincided with the depth of maximum methanotrophic activity (Fig. 1B, S3), suggesting that MOB were potentially major contributors to the CH_4_ oxidation in this lake. This is not surprising since the possible involvement of aerobic methanotrophs in anaerobic methane oxidation has been previously suggested^4,20,21^. Although MOB activity was not determined, this finding indicates that MOB might be active in anoxic waters. Quantitative PCR also revealed that the amount of *mcrA* gene was minimal in the MMZ and remained low where the methanotrophic peak was observed, being two orders of magnitude less abundant than the *pmoA* gene (Fig. 1B). This result suggests that *mcrA*-carrying anaerobic methane oxidizing archaea (ANME) were not major contributors to the strong methane oxidation observed in this anoxic layer. The *mcrA* gene abundance only increased significantly at the bottom of the water column (1.1 × 10^7^ ± 0.3 × 10^7^ *pmoA* copies L^−1^ at 10 m depth); similar results were observed with total archaea (quantified through their 16 S rRNA gene). In addition, a local *mcrA* maximum (2.3 × 10^5^ ± 0.4 × 10^5^ copies L^−1^) was observed in the oxic epilimnion at a depth of 2 m, which indicates the presence of methanogens despite the oxic environment.

The major CH_4_ oxidation observed in the anoxic epilimnion raises the important question of the oxic or anoxic nature of the process. Several arguments suggest the absence of oxygen, either diffused from the top or locally produced. First, the euphotic depth of 4.9–5.6 m was 2.9–3.6 m above the peak of methanotrophic activity, where the photosynthetic available radiation was 0.03–0.08% of its surface value. Second, the peak of methanotrophic activity of 1.82 ± 0.34 μmol CH_4_ L^−1^ h^−1^ would require 3.64 ± 0.68 μmol O_2_ L^−1^ h^−1^, i.e., two moles of O_2_ required to oxidize one mole of CH_4_. That oxygen requirement is within the higher range of primary production reported for the euphotic or epilimnion zones of 118 lakes^22^ worldwide, thus unlikely to occur 2.9–3.6 m below the euphotic depth. Third, the average methanotrophic activity over the entire water column, determined by integration of negative NMPR, corresponded to 611 ± 80 μmol CH_4_ m^−2^ h^−1^. That figure would require a counter flux of 1,222 ± 160 μmol O_2_ m^−2^ h^−1^. According to a maximum hypolimnetic diffusivity of 2.819 m^2^ h^−1^^23^, the DO gradient along the water column, required to sustain aerobic methanotrophy, corresponds to 433 μmol L^−1^ per meter of water column depth, which is incompatible with the measured DO concentration in the hypolimnion; i.e., below 0.3 μmol L^−1^. Fourth, the proximity of methanogenic peaks, observed 0.51 ± 0.11 m below the methanotrophic peak, does not support a sudden shift from an active oxic to a strict anaerobic process. The unlikeliness of aerobic CH_4_ oxidation raises the question of the electron acceptor as an alternative to O_2_. No evidence arises from nitrate or nitrite (Table S2), as none of these electron acceptors for anaerobic methane oxidation^20,24^ showed a significant concentration change in the region where methanotrophic activity was found, and both nitrate or nitrite in the lake water column were below or at the lower range of concentration to support AOM coupled to denitrification^25^. Thus, the present work does not reveal the possible electron acceptors for AOM in the epilimnion of the lake, since sulfate, oxidized metals, and organic matter^26^ were not tested.

Our results show that the MMZ segregates two different CH_4_ zones of the water column with no diffusive exchange between them. The existence of an MMZ segregation zone is supported not only by the CH_4_ profiles, discarding diffusive transfer between the epilimnion and the hypolimnion, but also by the CH_4_ isotopic signature, suggesting different CH_4_ origins in each zone. However, despite segregation between the epilimnion and the hypolimnion, bubbles formed in the sediments might transfer CH_4_ to the epilimnion during their migration to the surface of the lake. Nevertheless, at each sampling and monitoring station, CH_4_ and CO_2_ fluxes were determined in triplicate with a dynamic closed chamber coupled to a greenhouse gas analyzer (UGGA 30 P, Los Gatos Research, CA, USA; data acquisition frequency of 1 s^−1^ and CH_4_ sensitivity of 30 ppb)^17^. During a total of 54 flux measurements over a total time of 4.5 h, no evidence of bubbling was found; i.e., peak increase of CH_4_ concentration within the chamber^17^. Bubbles were only occasionally visually observed in the littoral region at the western section of the lake. Thus, ebullitive transfer of CH_4_ from the hypolimnion to the epilimnion, is unlikely although not discountable. The CH_4_ found at the epilimnion might be considered as the product of local oxic production and/or lateral transport from the littoral zone^1,8,10–13^.

**Figure 3:**
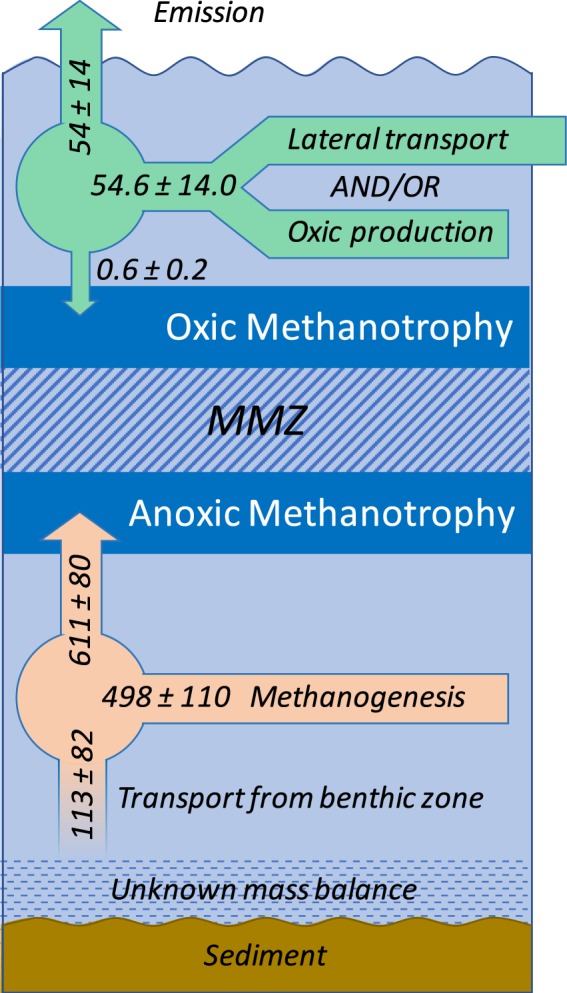
CH_4_ mass balance in the water column of Sila Lake. Arrows indicate CH_4_ transport and production while numbers indicate the magnitude of these processes, all of which are expressed per unit of lake area (μmol CH_4_ m^−2^ h^−1^). The minimum methane zone is called MMZ.

We estimated the flux, downward to the MMZ, as 0.62 ± 0.15 μmol CH_4_ m^−2^ h^−1^ (Fig. S3 A–C). A triplicate measurement of the CH_4_ flux to the atmosphere, at each of the three locations where the C_CH4_ profiles were made, gave an average of 54 ± 14 μmol CH_4_ m^−2^ h^−1^. Thus, the transfer of CH_4_ to the MMZ was negligible and, assuming steady-state concentrations (i.e., those that did not change over time), we estimated the oxic production and/or lateral transport to equalize flux to the atmosphere, i.e., 54 ± 14 μmol CH_4_ m^−2^ h^−1^. Figure 3 shows the CH_4_ mass balance of Sila Lake at the time of characterization and indicates that about 92% of the total CH_4_ produced in or transported to the lake is oxidized. The steady-state assumption used to establish the CH_4_ mass balance might be a simplistic consideration regarding the epilimnion. Sila Lake is a northern lake with an ice-free period ranging from three to four months (personal communication with local inhabitants); this short period of summer stratification suggests relatively rapid changes of the water column structure, which is potentially contradictory to steady-state conditions. However, the total amount of CH_4_ present in the epilimnion, considering a depth layer of 2.3 m, was estimated to be 1.34 ± 0.12 mmol CH_4_ m^−2^. Dividing the amount of CH_4_ present in the epilimnion by the flux to the atmosphere mentioned above, we estimated the CH_4_ turnover time to range from 22–27 h. The latter indicates that the CH_4_ cycle in the epilimnion is dynamic and that the CH_4_ emitted to the atmosphere is rapidly replaced by lateral transport and/or oxic production.

This study shows that, in at least some cases, CH_4_ cycling in stratified lakes should be considered as two segregated systems with no exchange between them. While the hypolimnion is dominated by methanotrophy and hydrogenotrophic methanogenesis, the epilimnion is dominated by CH_4_ originating from acetoclastic production, *in situ* or transferred from surrounding terrestrial ecosystems. The segregation between the CH_4_ cycle in the epilimnion and the hypolimnion has been suggested by several reports^5,8,10,12,13^ and used as a supporting evidence that epilimnetic CH_4_ is locally produced^8,10–13,27^ or transported from the littoral zone^1,8,28^. In addition to similar conclusions, the present work was based on an innovative high resolution and sensitive method for determining C_CH4_, which allowed the NMPR profile to be created and demonstrated that methanotrophy can occur well below the oxycline and euphotic zone of a lake. This method also revealed the drastic shift, relative to depth, between the two opposing dominant CH_4_ processes of anaerobic methanogenesis and methanotrophy, with the latter being potentially anoxic or at least not associated with locally produced oxygen. Notably, *pmoA*-carrying bacteria were the major microbial contributors of methane oxidation in this lake and largely predominated (over potential ANMEs) at the depth of highest methane oxidation activity, leading to a complete exhaustion of produced methane at the hypolimnetic water column. The extent to which the behavior observed in Sila Lake can be extrapolated to other lakes is unknown but is certainly an important milestone and should be investigated further, along with the identity of the methane oxidizing bacteria potentially involved in AOM in this lake and others.

## Materials and Methods

A small, Y-shaped unnamed lake, located in a discontinuous permafrost area of northern Siberia^29^ 5 km north of the town of Igarka, Krasnoyarsk Kray, Russia (Fig. S1), was selected and called Sila for the purpose of this study (Lat. 67.5138, Long. 86.5915). Sila Lake is of glacial origin and heavily influenced by thermokarst. It is surrounded by northern forest and peatlands, which is the dominant landscape of the region. About one-third of the perimeter (600 m), mostly on the west, is bordered by shallow peatland, while mixed forest, i.e., larch (*Larix sibirica*), birch (*Betula pendula*), and Siberian pine (*Pinus sibirica*) borders about two-thirds of the perimeter (1100 m). In August 2016, sampling and monitoring stations were established at 13 locations along a west-east longitudinal transect and 5 locations along a transversal north-south transect (Fig. S1). C_CH4_ and C_CO2_ concentrations along the water column were determined using a membrane-integrated cavity output spectrometry method (M-ICOS)^16^. This method, described in more detail in the supporting information, allowed for the continuous measurement of dissolved gas at a frequency of 1 s^−1^, which corresponded to approximately 50 dissolved gas concentration data points per meter of the water column. The lower detection limit of the method under the present configuration was 5 nmol L^−1^ for C_CH4_ and 4 μmol L^−1^ for C_CO2_. Vertical CH_4_ fluxes and NMPR within the water column were derived from the estimation of turbulent diffusion of CH_4_ across the concentration gradient according to the method established in Kankaala *et al*.;^30^ details of the method are provided in the supporting information. At each location, water column parameters including pH, temperature, DO, redox potential, and conductivity were also determined at 1 m depth intervals using multi-parametric probes (HI 9828, Hanna Instrument, Mexico). Water transparency was measured with a 30 cm Secchi disk. The euphotic depth (Z_1%_) was estimated according to methods established by French *et al*.^31^ and LaPierre and Edmundson^32^.

At the deepest location, water samples were taken at 1, 2, 4, 5, 6, 8, 9, and 10 m with a Van Dorn Bottle. These samples were used for molecular biology, stable isotope analysis of dissolved inorganic carbon (δ^13^C-DIC) and CH_4_ (δ^2^H-CH_4_ and δ^13^C-CH_4_). All stable isotope samples were analyzed in replicates of three and standard deviation was typically 0.2‰. The δ^13^C-DIC was analyzed by an Isoprime 100 unit (MultiFlow-Geo, Elementar, UK). Stable isotopic analysis of CH_4_ (δ^2^H and δ^13^C) was completed at UC Davis’ Stable Isotope Facility with a Thermo Scientific PreCon unit interfaced to a Thermo Scientific Delta V Plus Isotope Ratio Mass Spectrometer (Bremen, Germany). The fractionation factor α indicates the magnitude of isotopic separation between the δ^13^C values of ΣCO_2_ (total dissolved inorganic carbon) and CH_4_ in anaerobic environments. Its value reflects the dominant CH_4_ production pathways. It also shows systematic shifts in CH_4_ oxidation processes^33^. It was calculated as: α = (δ^13^C-DIC + 1000)/(δ^13^C-CH_4_ + 1000)^18^. For molecular biology characterization, water samples were filtered as soon as possible after sampling (i.e. <24 h). DNA extraction was completed and quantitative PCR (qPCR) was performed to assess the abundance of the following genes: bacterial 16 S rRNA gene (total bacteria), archaeal 16 S rRNA gene (total archaea), *pmoA* gene (particulate methane monooxygenase) and *mcrA* gene (methyl coenzyme M reductase, indicative of methanogens and ANMEs). More details of these methods are provided in the supporting information.

## Supplementary information

Supplementary Information.

## Data Availability

The data analyzed in this study are available from the corresponding author upon request.
