# Supplementary material for: Sub-oxycline methane oxidation can fully uptake CH4 produced in sediments: case study of a lake in Siberia
Source: Sci Rep. 2020 Feb 25;10:3423. doi: 10.1038/s41598-020-60394-8 (PMC7042212; doi:10.1038/s41598-020-60394-8)
Supplement: Supplementary file 1 — Supplementary Information. [file 41598_2020_60394_MOESM1_ESM.docx]

**Sub-oxycline methane oxidation can fully uptake CH_4_ produced in the sediments: case study of a Siberian lake**

Frédéric Thalasso^1,2^, Armando Sepulveda-Jauregui^*,2,3^, Laure Gandois^4^, Karla Martinez-Cruz^2^, Oscar Gerardo-Nieto^1^, María S. Astorga-España^2^, Roman Teisserenc^4^, Céline Lavergne^5^, Nikita Tananaev^6^, Maialen Barret^4^, & Léa Cabrol^7^

^1^Biotechnology and Bioengineering Department, Center for Research and Advanced Studies (Cinvestav), Mexico City, Mexico

^2^The Environmental Biogeochemistry in Extreme Ecosystems Laboratory (EnBEELab), University of Magallanes, Punta Arenas, Chile

^3^Center for Climate and Resilience Research (CR)^2^, Santiago, Chile

^4^EcoLab, Université de Toulouse, CNRS, Toulouse, France

^5^Escuela de Ingeniería Bioquímica, Pontificia Universidad de Valparaiso, Valparaiso, Chile

^6^Melnikov Permafrost Institute, Yakutsk, Russia

^7^Aix-Marseille University, Univ Toulon, CNRS, IRD, Mediterranean Institute of Oceanography, Marseille, France

^*^Correspondence and requests for materials should be addressed to A. S-J, email: armando.sepulveda@umag.cl; Av. Manuel Bulnes 01890, Punta Arenas, Chile; Phone: +56984877395; Fax: +5661212835.

**Supporting information**

**Material and Methods**

M-ICOS method

M-ICOS is a membrane equilibration method, in which a continuous flow of water (1 L min^–1^) is extracted at the desired depth from the freshwater ecosystems through a peristaltic pump and forced to flow through the shell of a gas-liquid exchange module (Permselect PDMSXA-1000, Medarray Inc., USA). Inside the hollow fibers of the membrane module (1250 silicone hollow fibers of 190 μm internal diameter), a continuous flow (2 L min^–1^) of CH_4_- and CO_2_-free nitrogen is distributed, controlled by a mass flow controller (GFC17, Aalborg, USA). The nitrogen flowing out of the gas-liquid exchange module is then measured with a CH_4_ and CO_2_ analyzer, in this case an OA-ICOS ultraportable greenhouse gas analyzer (UGGA, model 915-0011, Los Gatos Research Inc., USA). Since the mass transfer through the silicone membranes is proportional to the concentration of the dissolved CH_4_ and CO_2_ concentration in the sampled water, their concentrations can be determined, after proper calibration. Details of the method can be found elsewhere.^1^

In Sila Lake, the water was extracted using a weight-probe (200 g) containing a water filter and connected to a polyurethane tubing (4 mm internal diameter, Festo, Mexico). Prior to use, the method was calibrated, which included (i) the determination of the delay time; i.e., delay between the time the water is actually extracted by the probe and the time it reaches the gas/liquid module, (ii) the response time; i.e., response time of the membrane module and the gas analyzer, and (iii) the gas transfer coefficient, which establishes the proportionality between the dissolved gas concentration and the concentration read by the analyzer. The gas transfer coefficient was determined using a standard headspace equilibration method with samples taken from the epilimnion and the hypolimnion, to cover a range of CH_4_ and CO_2_ concentrations. After calibration, the profile procedure was as follows: the probe was maintained a few centimeters below the water surface for about 1 min; then the probe was lowered slowly and steadily by a trained operator, to the bottom of the lake, where it was maintained for an additional minute. A controlled diving speed was maintained and to know the depth corresponding to each concentration data, the time at each 1 m intermediary depths was noted and used to determine intermediary depth. With this procedure, we estimated that no depth determination error was committed at each 1 m intermediary depths and that a maximum error of 13% was committed on the decimal fraction of the depth, in the middle between two intermediary depths; e.g., 0.5, 1.5, 2.5… m. The diving speed was about 1.2 m min^–1^ ± 13% and therefore about 50 data were acquired for each m of water column depth.

Diffusion model

Vertical CH_4_ fluxes and net methane production rates (NMPR), within the water column, were derived from the estimation of turbulent diffusion of CH_4_ across the concentration gradient in the water column, according to Kankaala et al.^2^ Briefly, the turbulent diffusion coefficient K (m^2^ d^–1^) was estimated from Eq. S1:

$K=0.00706\cdot A^{0.56}\cdot\left( N^{2} \right)^{-0.43}$, (S1)

where A is the lake surface area (0.036 km^2^), and N^2^ is the Brunt-Väisälä stability frequency (S^–2^),^3^ defined by Eq. S2, as follows;

$N^{2}=\frac{g}{\rho}\cdot\frac{\partial\rho}{\partial z}$, (S2)

where g is the gravitational constant (9.81 m^2^ s^–1^), ρ is the density of water, determined from Tanaka et al,^4^ (kg m^–3^) and z is the depth (m). A minimum possible stability frequency of 7.0 10^–5^ s^–2^ was considered, which sets the upper limit for K to 67.6 10^–3^ m^2^ d^–1^. This was the case of the water column of Sila Lake at depth > 4.5 m.

Flux entering each segment of the water column; i.e., distance (∂z) between two intermediary dissolved gas data multiplied by an arbitrary area of 1 m^2^, was determined according to the Fick’s equation (Eq. S3);

$F=\frac{K}{24}\cdot\frac{\partial C}{\partial z}$, (S3)

where F is the flux (mole m^–2^ h^–1^), 24 is the unit conversion of K, from m^2^ d^–1^ to m^2^ h^–1^, and C_CH4_ is the dissolved CH_4_ concentration (mole m^-3^). Despite the high signal to noise ratio of the UGGA detector; i.e., ratio of the mean to the standard deviation, reported to 1520 ± 415,^1^ fluxes were subject to a significant noise, and a data smoothening of C_CH4_ was necessary. We opted for a pondered smoothening described by Equation S4, where X´ is the smoothened variable X, in this case C_CH4_, and z denotes the depth of measurement.

$X_{z}^{'}=0.1\cdot X_{z-2}+0.2\cdot X_{z-1}+0.4\cdot X_{z}+0.2\cdot X_{z+1}+0.1\cdot X_{z+2}$ (S4)

Next, the NMPR was determined in each segment of the water column from the difference between flux entering and exiting the water column segment. To reduce noise, fluxes used to determine NMPR were smoothened, and then NMPR was smoothened again, in both cases using Eq. S4. We acknowledge that this method does not distinguish between actual methane production/oxidation; i.e., methanogenesis/methanotrophy, and methane input/output by lateral transport.

Molecular biology

Water samples were pre-filtered at 80 µm (nylon net filters, Merck Millipore, Ireland) and filtered at 0.22 µm (nitrocellulose GSWP membrane filters, Merck Millipore, Ireland) until clogging (i.e. corresponding to 350 to 1180 mL of filtered volume, depending on sample characteristics). Filters were stored at –20 °C and used for DNA extraction using the PowerWater DNA isolation kit (Qiagen). DNA quantity and quality were checked by spectrophotometry. Quantitative PCR (qPCR) was performed to assess the abundance of the following genes: bacterial 16S rRNA gene (total bacteria), archaeal 16S rRNA gene (total archaea), *pmoA* gene (particulate methane monooxygenase) and *mcrA* gene (methyl coenzyme M reductase, indicative of methanogens and ANMEs). All qPCRs were carried out in duplicate of 20 µL, with Takyon SYBR master mix (Eurogentec), on CFX96 thermocycler (Bio-Rad Laboratories), using 100-fold diluted template DNA (approximately 0.4 ng). For bacterial and archaeal 16S rRNA gene and *mcrA*, standard curves were prepared from 10-fold serial dilutions of each target gene, amplified from pure strains; i.e., *Pseudomonas stutzeri* SLG510A3-8, Arch-_21F_10 clone KT351355, and *Methanosarcina barkeri* CM1, respectively, and cloned in pGEM-T plasmid (Promega). For *pmoA*, the standard was synthetized by Eurofins from *Methylobacter* sp. BB5.1 *pmoA* gene sequence, inserted in TOPO-TA pCR2.1 plasmid. qPCR conditions as well as primer sequences and concentrations are detailed in Table S1. qPCR efficiencies were always > 90% and amplicon size and unicity were confirmed by melting curve analysis and agarose gels.

**Table S1.** qPCR primers and conditions used in this study. The thermocycler program was as follows: initial denaturation (95 °C, 3 min), 40 cycles of denaturation (95 °C, 10 s), hybridization (specified in the Table), elongation (72 °C, 30 s). For *mcrA* amplification, an extra step was added (80 °C, 8 s) to deal with highly degenerated primers.

| **Target gene** | **Primers** | **Sequence (5’-3’)** | **Hybridization conditions** | **Final conc. (µM)** | **Amplicon length (bp)** | **References** |
| --- | --- | --- | --- | --- | --- | --- |
| Bacterial 16S rRNA | GML5F  Univ516R | GCCTACGGGAGGCAGCAG  GTDTTACCGCGGCKGCTGRCA | 55 °C, 20 s | 0.4 | 172 | Muyzer et al.,^5^ Takai and Horikoshi^6^ |
| Archaeal 16S rRNA | 931F  m1100R | AGGAATTGGCGGGGGAGCA  BTGGGTCTCGCTCGTTRCC | 62 °C, 20 s | 0.4 | 169 | Jackson et al.,^7^ Einen et al.^8^ |
| *mcrA* | mlas  mcrA-rev | GGTGGTGTMGGDTTCACMCARTA  CGTTCATBGCGTAGTTVGGRTAGT | 55 °C, 20 s | 1 | 470 | Steinberg and Regan^9^ |
| *pmoA* | A189F  mb661R | GGNGACTGGGACTTCTGG  CCGGMGCAACGTCYTTACC | 55 °C, 20 s | 0.4 | 508 | Holmes et al.,^10^ Kolb et al.^11^ |

Physicochemical and isotopic characterization

Non-purgeable organic carbon (NPOC), referred as Dissolved Organic Carbon (DOC), were determined from filtered samples, kept in glass vials and acidified to pH 2 prior to analysis. DOC concentration was determined with a TOC-V CSH analyzer (Shimadzu, Japan), with a detection limit of 1 mg L^–1^. The δ^13^C-DIC was analyzed on water samples kept in glass vials, poisoned with HgCl_2_, acidified using phosphoric acid and flushed with helium, previous to mass spectrometry analyzer (Isoprime 100, Elementar, UK) coupled to an equilibration system (MultiFlow-Geo, Elementar, UK). Standards included Na_2_CO_3_ and NaHCO_3_ as well as internal water standards. All standards were analyzed every eight samples to check for instrument stability. All samples were analyzed in two replicates. Standard deviation was typically 0.2 ‰. Stable isotopic analysis of CH_4_ (δ^2^H-CH_4_ and δ^13^C-CH_4_) were determined at UC Davis stable isotope facility (https://stableisotopefacility.ucdavis.edu/ch4.html). With that purpose, water samples were collected in air-tight glass vials and acidified in the field using ultra-pure HCl. Prior to measurement, dissolved CH_4_ was extracted for IRMS analysis following Yarnes.^12^ Samples were measured with a ThermoScientific Precon concentration unit interfaced to a ThermoScientific Delta V Plus isotope ratio mass spectrometer (ThermoScientific, Bremen, Germany). The detection limit was 5 ppm of CH_4_ for δ^2^H and 1.7 ppm of CH_4_ for δ^13^C, and standard deviation was typically 2 ‰ for δ^2^H and 0.2 ‰ for δ^13^C. The fractionation factor α was calculated using the δ^13^C-DIC and δ^13^C-CH_4_ as follow : α=( δ^13^C-DIC+1000)/( δ^13^C- CH_4_ +1000).^13^

**Results**

*Figure S1: Bathymetric map of Sila Lake, showing surrounding ecosystems. Sampling station marked in red (P1) shows the location where triplicate C_CH4_ and C_CO2_ profiles were determined (Fig. 1). ND, not determined.*

*Figure S2: Temperature (red triangles) and dissolved oxygen concentration (blue circles) over the water column of Sila Lake.*

*Figure S3: Triplicate CH_4_ flux profiles (A-C) and NMPR (D-F) profiles. Epilimnion fluxes, marked in green, are multiplied by 1000, while epilimnion NMPR, also marked in green, are multiplied by 100.*

**Table S2**: Main physicochemical parameters measured at location P1 in lake Sila.

| Depth  (m) | Conductivity  (mS cm^–2^) | pH  (–) | DOC^A^  (mg L^–1^) | DIC^B^  (mg L^–1^) | N-NO_2_^–^  (mg L^–1^) | N-NO_3_^–^  (mg L^–1^) |
| --- | --- | --- | --- | --- | --- | --- |
| 0 | 89.0 | 7.02 | -^C^ | 3.67 | - | - |
| 1 | 90.0 | 7.11 | 8.75 | 3.08 | - | 0.0030 |
| 2 | 90.0 | 7.09 | 8.66 | 3.00 | 0.0184 | 0.0020 |
| 4 | 106.0 | 6.90 | 8.01 | 3.46 | 0.0195 | 0.0020 |
| 5 | 111.0 | 6.73 | 8.28 | 3.44 | 0.0193 | 0.0070 |
| 6 | 117.0 | 6.57 | 8.43 | 4.39 | 0.0187 | 0.0040 |
| 8 | 130.0 | 6.49 | 7.61 | 4.76 | 0.0176 | 0.0010 |
| 9 | 193.0 | 7.46 | 9.07 | 7.17 | 0.0189 | 0.0030 |
| 10.5 | 205.0 | 7.56 | 8.42 | 10.24 | 0.0203 | 0.0040 |

*^A^ Dissolved Organic Carbon; ^B^ Dissolved Inorganic Carbon; ^C^ - not determined.*

**References**

1. Gonzalez-Valencia, R. *et al.* In Situ Measurement of Dissolved Methane and Carbon Dioxide in Freshwater Ecosystems by Off-Axis Integrated Cavity Output Spectroscopy. *Environ. Sci. Technol.* **48**, 11421–11428 (2014).

2. Kankaala, P., Huotari, J., Peltomaa, E., Saloranta, T. & Ojala, A. Methanotrophic activity in relation to methane efflux and total heterotrophic bacterial production in a stratified, humic, boreal lake. *Limnol. Oceanogr.* **51**, 1195–1204 (2006).

3. Hondzo, M. & Stefan, H. G. Lake Water Temperature Simulation Model. *J. Hydraul. Eng.* **119**, 1251–1273 (1993).

4. Tanaka, M., Girard, G., Davis, R., Peuto, A. & Bignell, N. Recommended table for the density of water between 0  C and 40  C based on recent experimental reports. *Metrologia* **38**, 301–309 (2001).

5. Muyzer, G., de Waal, E. C. & Uitterlinden, A. G. Profiling of complex microbial populations by denaturing gradient gel electrophoresis analysis of polymerase chain reaction-amplified genes coding for 16S rRNA. *Appl. Environ. Microbiol.* **59**, 695–700 (1993).

6. Takai, K. & Horikoshi, K. Rapid detection and quantification of members of the archaeal community by quantitative PCR using fluorogenic probes. *Appl. Environ. Microbiol.* **66**, 5066–72 (2000).

7. Jackson, C. R., Langner, H. W., Donahoe-Christiansen, J., Inskeep, W. P. & McDermott, T. R. Molecular analysis of microbial community structure in an arsenite-oxidizing acidic thermal spring. *Environ. Microbiol.* **3**, 532–42 (2001).

8. Einen, J., Thorseth, I. H. & Øvreås, L. Enumeration of Archaea and Bacteria in seafloor basalt using real-time quantitative PCR and fluorescence microscopy. *FEMS Microbiol. Lett.* **282**, 182–187 (2008).

9. Steinberg, L. M. & Regan, J. M. Applied and Environmental Microbiology. *Appl. Environ. Microbiol.* **60**, 3112–3119 (2008).

10. Holmes, A. J. *et al.* Characterization of methanotrophic bacterial populations in soils showing atmospheric methane uptake. *Appl. Environ. Microbiol.* **65**, 3312–8 (1999).

11. Kolb, S., Knief, C., Stubner, S. & Conrad, R. Quantitative detection of methanotrophs in soil by novel pmoA-targeted real-time PCR assays. *Appl. Environ. Microbiol.* **69**, 2423–9 (2003).

12. Yarnes, C. δ ^13^ C and δ ^2^ H measurement of methane from ecological and geological sources by gas chromatography/combustion/pyrolysis isotope-ratio mass spectrometry. *Rapid Commun. Mass Spectrom.* **27**, 1036–1044 (2013).

13. Blaser, M. & Conrad, R. Stable carbon isotope fractionation as tracer of carbon cycling in anoxic soil ecosystems. *Curr. Opin. Biotechnol.* **41**, 122–129 (2016).
